# Cytoprotective Co-chaperone BcBAG1 Is a Component for Fungal Development, Virulence, and Unfolded Protein Response (UPR) of *Botrytis cinerea*

**DOI:** 10.3389/fmicb.2019.00685

**Published:** 2019-04-09

**Authors:** Honghong Zhang, Yurong Li, Martin B. Dickman, Zonghua Wang

**Affiliations:** ^1^Fujian University Key Laboratory for Plant-Microbe Interaction, College of Plant Protection, Fujian Agriculture and Forestry University, Fuzhou, China; ^2^Institute for Plant Genomics and Biotechnology, College of Agriculture and Life Sciences, Texas A&M University, College Station, TX, United States; ^3^Department of Plant Pathology and Microbiology, College of Agriculture and Life Sciences, Texas A&M University, College Station, TX, United States; ^4^Institute of Oceanography, Minjiang University, Fuzhou, China

**Keywords:** BAG protein, co-chaperone, *Botrytis cinerea*, pathogenicity/virulence, unfolded protein response

## Abstract

The Bcl-2 associated athanogene (BAG) family is an evolutionarily conserved group of co-chaperones that confers stress protection against a variety of cellular insults extending from yeasts, plants to humans. Little is known, however, regarding the biological role of BAG proteins in phytopathogenic fungi. Here, we identified the unique BAG gene (*BcBAG1*) from the necrotrophic fungal pathogen, *Botrytis cinerea*. BcBAG1 is the homolog of *Arabidopsis thaliana* AtBAG4, and ectopic expression of *BcBAG1* in *atbag4* knock-out mutants restores salt tolerance. *BcBAG1* deletion mutants (Δ*Bcbag1*) exhibited decreased conidiation, enhanced melanin accumulation and lost the ability to develop sclerotia. Also, *BcBAG1* disruption blocked fungal conidial germination and successful penetration, leading to a reduced virulence in host plants. BcBAG1 contains BAG (BD) domain at C-terminus and ubiquitin-like (UBL) domain at N-terminus. Complementation assays indicated that BD can largely restored pathogenicity of Δ*Bcbag1*. Abiotic stress assays showed Δ*Bcbag1* was more sensitive than the wild-type strain to NaCl, calcofluor white, SDS, tunicamycin, dithiothreitol (DTT), heat and cold stress, suggesting BcBAG1 plays a cytoprotective role during salt stress, cell wall stress, and ER stress. BcBAG1 negatively regulated the expression of *BcBIP1*, *BcIRE1* and the splicing of *BcHAC1* mRNA, which are core regulators of unfolded protein response (UPR) during ER stress. Moreover, BcBAG1 interacted with HSP70-type chaperones, BcBIP1 and BcSKS2. In summary, this work demonstrates that BcBAG1 is pleiotropic and not only essential for fungal development, hyphal melanization, and virulence, but also required for response to multiple abiotic stresses and UPR pathway of *B. cinerea*.

## Introduction

Co-chaperones are proteins that assist chaperones in protein folding, oligomeric assembly, and protein transportation and degradation (Hartl, 1996). The Bcl-2-associated athanogene (BAG) family is a group of broadly conserved co-chaperones of 70-kilodalton heat shock protein (HSP70) (Bracher and Verghese, 2015). In mammals, the BAG family was initially identified by screening mouse cDNA library for Bcl-2 interaction proteins (Takayama et al., 1995). Using the ATPase domain of HSC70/HSP70 as molecular bait in yeast two-hybrid screening, additional BAG family members were identified from human, *Caenorhabditis elegans* and the fission yeast *Schizosaccharomyces pombe* (Takayama et al., 1999). The human BAGs contain six members, BAG1 to BAG6, and share a conserved BAG domain (BD) of approximately 45 amino acids, located near the C terminus (Takayama and Reed, 2001). As nucleotide exchange factors (NEFs) of HSP70, BAG proteins play a major role in both positively and negatively modulating HSP70 ATP activity via the BAG domain (Gassler et al., 2001). Moreover, BAGs act as scaffolds between HSP70 and target transcription factors or proteins, thus affecting diverse physiological events (Townsend et al., 2003; Kabbage and Dickman, 2008).

The identification and preliminary characterization of plant BAG proteins is still underway. Using a combination of bioinformatics and structural algorithms, seven BAGs have been identified from *Arabidopsis thaliana* (AtBAG1–AtBAG7) (Doukhanina et al., 2006), several of which have been selected for functional characterization. The structural and biochemical data of AtBAGs demonstrate that the AtBAGs function as NEFs for HSP70/HSC70 and the regulation mechanism of HSP70/HSC7 is conserved in plants (Fang et al., 2013). Our previous work has uncovered that AtBAG4, 6, and 7 exhibit different cytoprotective specificities. Briefly, AtBAG4 appears to protect plants from various abiotic stress stimuli, e.g., salt and drought (Doukhanina et al., 2006). AtBAG6 is activated via proteolytic cleavage by a specific plant aspartyl protease that is required for autophagic cell death *in planta* and subsequent resistance to the necrotrophic fungus *Botrytis cinerea* (Kabbage et al., 2016; Li and Dickman, 2016; Li et al., 2016). Consistently, overexpression of AtBAG6 induced programmed cell death (PCD) in both yeast and plants (Kang et al., 2006). The ER-localized AtBAG7 is an essential component of the unfolded protein response (UPR) and directly interacts with UPR regulator AtBIP2 (Williams et al., 2010). Under heat stress, AtBAG7 is also proteolytically processed in the ER lumen and translocate from the ER to the nucleus, where it interacts with the transcriptional factor WRKY29 for heat tolerance (Li et al., 2017). A number of BAGs have also been reported in other plant species. For example, OsBAG4 from rice interacts with an E3 ubiquitin ligase EBR1 and regulates PCD, which controls plant immunity and broad-spectrum disease resistance (You et al., 2016). Taken together, plant BAGs are multifunctional and modulate numerous physiological and biological processes.

Although the functions of human and plants BAG family members are extensively studied, there is limited knowledge on the roles of the BAGs in fungi. Previous studies showed that SNL1, a mammalian homolog of BAG1 in *Saccharomyces cerevisiae*, is functionally linked to the nuclear pore complex and plays a role in promoting both protein biogenesis and translation by recruiting ribosomes and HSP70 to the ER membrane (Ho et al., 1998; Verghese and Morano, 2012). Two other BAG1 homologs from *Schizosaccharomyces pombe*, *BAG101* and *BAG102*, are co-factors of 26S proteasomes, and play as HSP70 chaperones (Kriegenburg et al., 2014). Overexpression of *BAG101* and *BAG102* inhibit cell growth by triggering HSP70 to release and activate HSF1 (heat shock factor 1) (Poulsen et al., 2017). To date, the only example in filamentous fungi is BAGA from *Aspergillus nidulans*, which impacts fungal sexual development and modulates secondary metabolism (Jain et al., 2018).

The endoplasmic reticulum (ER) is the central intracellular organelle for protein translocation, protein folding, and protein post-translational modifications, allowing further transport of proteins to the Golgi apparatus and ultimately to vesicles for secretion or display on the plasma surface. Perturbations in ER function, named “ER-stress,” unfolded or misfolded proteins accumulate within the ER and disrupt ER homeostasis to activate an intracellular signaling pathway, known as the UPR eventually culminating in cell death (Malhotra and Kaufman, 2007). As a conserved survival pathway to counteract the lethal effects caused by ER stress, UPR can mitigate accumulation of unfolded proteins and restore ER homeostasis by reducing protein translation and while increasing and misfolded proteins degradation aided by molecular chaperones (e.g., binding immunoglobulin proteins, BiPs) (Sitia and Braakman, 2003; Ron and Walter, 2007). BiPs work as HSP70 chaperones and carry aberrant proteins from the ER to the cytoplasm for degradation by the proteasome (Gething, 1999). The IRE1-HAC1/XBP-1 pathway (HAC1 mRNA in yeast and XBP1 mRNA in metazoans) is a major branch of UPR that is remarkably conserved from yeast to human (Back et al., 2005). UPR is initiated by the activation of the ER stress sensor IRE1, which transmits the signal by removing a non-conventional intron from a transcription factor HAC1/XBP-1 mRNA to produce potent transcriptional activator of UPR targets (Ron and Walter, 2007). Comparing to the extensive studies of UPR in human and plant systems, UPR has only been delineated in small number of fungal pathogens, including *Aspergillus fumigatus* (Feng et al., 2011), *Alternaria brassicicola* (Joubert et al., 2011), *Ustilago maydis* (Heimel et al., 2013; Lo Presti et al., 2016), and all of which demonstrate that UPR regulation is correlated with fungal pathogenicity.

In this study, we identified a unique *BAG* gene in the necrotrophic fungal pathogen *Botrytis cinerea*, the causal agent of gray mold diseases to over 1,400 species of cultivated plants worldwide (Elad, 2016). Target gene replacement of *BcBAG1* resulted to defect in vegetative growth, conidiation, sclerotial formation, penetration and attenuated virulence in *B. cinerea*, Δ*Bcbag1* mutants were more sensitive to various stress conditions indicating that BcBAG1 regulates stress tolerance in *B. cinerea*. In particular, *BcBAG1* deletion mutants significantly increased susceptibility to diverse ER stress-inducer including heat, cold, tunicamycin (Tm), and dithiothreitol (DTT). We demonstrated that BcBAG1 binds to the ER chaperone BcBIP1 and negatively regulate UPR components, including the expression of *BcBIP1*, *BcIRE1* and the splicing of *BcHAC1* mRNA, suggesting BcBAG1 is necessary for the maintenance of the UPR in *B. cinerea*. Collectively, we present the evidence of identification and functional characterization for BcBAG1 a member of BAG family in *B. cinerea*, which is vital for fungal virulence on host plants and is required for ER stress response with regards to maintenance of UPR.

## Materials and Methods

### Strains and Culture Conditions

*BcBAG1* deletion mutant, Δ*Bcbag1*, was generated from the *B. cinerea* WT strain B05.10 (Quidde et al., 1999). All strains (Supplementary Table S1) were maintained on potato dextrose agar (PDA) medium at 22°C. Mycelia used for protoplast preparation, genomic DNA and total RNA extraction were grown in YEPD (1% peptone, 0.3% yeast extract, 2% glucose, pH 6.7) at 150 rpm, 22°C for 36 h. *B. cinerea* protoplast was recovered on SH medium (20% sucrose, 0.5 mM hepes, 1 mM NH_4_H_2_PO_4_, pH 7.0) at 22°C for 12–16 h. The selectable marker, 100 μg/ml hygromycin B (VWR) or 100 μg/ml nourseothricin sulfate (Research Products International) was supplemented to PDA containing 1% agar.

### Bioinformatic Analysis

Preliminary BAG protein search and DNA sequence downloading were conducted in *B. cinerea* B05.10 genome database^1^. The phylogenetic tree was generated through MEGA v7.0 based on the neighbor-joining method (Kumar et al., 2016). Domain is predicted by Pfam^2^ and InterPro programs^3^. The multiple alignment of BAG domain sequence were constructed using Clustal X (Larkin et al., 2007).

### *BcBAG1* Gene Deletion and Complementation

Primers used in this study are listed in Supplementary Table S2. The replacement constructs for *BcBAG1* were generated through the split-marker approach as described before (Goswami, 2012). Briefly, the 800-bp upstream and 855-bp downstream fragments of *BcBAG1* were amplified with primer pairs AF/AR and BF/BR (Supplementary Table S2), respectively. The resulting amplicons ligated with the hygromycin phosphotransferase (*hph*) fragments by using Splicing Overlap Extension (SOE)-PCR. The resulting PCR products (20 μg) were transformed into protoplasts of *B. cinerea.* Protoplast preparation and PEG-mediated transformation of *B. cinerea* were performed as the established protocol (Gronover et al., 2001). After transformation, hygromycin-resistant transformants were picked individually and PCR analyses with designated primer pairs OF/OR, UF/UR, and AF/BR were performed to identify transformants that carried the insertion of *hph* at the *BcBAG1* locus. Then all positive transformants were confirmed by subsequent RT-PCR and Southern blotting.

The full length of *BcBAG1* was amplified from B05.10 genomic DNA, then ligated into pNAH-OGG with *NcoI* to create *BcBAG1-GFP*. GFP-fusion constructs were transformed into B05.10 for subcellular localization analysis. For complementation, the CDS of *BcBAG1* with native promoter sequence were amplified with relative primer pairs (CF/CR) and cloned into pNAH-OMG harboring nourseothricin acetyltransferase gene (*NAT1*) with *SpeI*/*NcoI* to make *BcBAG1-Com*, conferring resistance to antibiotics nourseothricin sulfate. Similar method was used to create the truncated BcBAG1A^1-141^ and BcBAG1B^142-298^ constructs. These constructs were transformed into Δ*Bcbag1* mutants. Transformants with resistance to both nourseothricin and hygromycin were selected and confirmed by PCR and RT-PCR.

### Southern Blotting and Real-Time PCR

Fungal genomic DNA was extracted as described (Raeder and Broda, 1985). For Southern blotting, the genomic DNAs were digested with *PvuI* (NEB) for 24 h at 37°C. Probe labeling, hybridization and detection were performed in accordance with the manufacturer’s instructions for the Digoxigenin High Prime DNA Labeling and Detection Starter Kit I (Roche Applied Science).

Total RNA was isolated using Eastep^TM^ Total RNA extraction Kit, following the manufacturer recommendation (Corp). The first-strand cDNA was synthesized with the M-MLV (Moloney Murine Leukemia Virus) reverse transcriptase (Life Technologies). Quantitative RT-PCR (qRT-PCR) was performed with SYBR Green PCR master mix (Applied Biosystems). The fungal actin gene was used as an internal reference. The relative expression levels were calculated by the 2^-ΔΔCt^ method (Livak and Schmittgen, 2001).

### Mycelial Growth, Conidiation, and Sclerotial Formation Tests

For vegetative growth assays, 5 mm diameter mycelial plugs were cultured on fresh PDA in the dark at 22°C. Radial growth was measured by colony diameters after 3 days. Determination of the sensitivity of Δ*Bcbag1* to environmental stresses were performed on modified PDA plates with: 1 M NaCl, 1.2 M D-sorbitol, 2 μg/ml Tm, 2.5 mM DTT, 20 mM H_2_O_2_, 0.5 mM tert-butyl hydroperoxide (TBHP), 15 μM bortezomib (Bort), 200 μM MG132, 600 μg/ml calcofluor white (CFW), 0.02% SDS, 1 μg/ml iprodione (Ipro), 0.1 μg/ml fludioxonil (Flud). The percentage of mycelial radial growth inhibition (RGI) was calculated using the formula RGI% = [(*C* -*N*)/*C* ^∗^ 100], where *C* is colony diameter of the control, and *N* is colony diameter of the experimental treatment.

For conidiation assays, conidia of WT and mutants were collected from a 10-day-old PDA plate with 5 ml sterile water and spores were counted microscopically with a hemocytometer. For sclerotial formation, 5 mm diameter mycelial plugs were inoculated to PDA and incubated in the dark at 22°C, the number of mature (melanized) sclerotia were counted after 4 weeks.

### Conidial Germination and Fungal Penetration Assays

Conidial germination was conducted as described by Doehlemann et al. (2006). Briefly, conidial suspensions were adjusted to 1.0–1.5 × 10^5^ spores/ml in 10 mM KH_2_PO_4_ and 10 mM glucose solution and placed in the center of a glass slide. Incubation was kept in a moist chamber at 22°C for 12 h, 24 h, and 48 h. For the onion infection assay, 20 μl droplets (5.0 × 10^4^ spores/ml) were deposited on the hydrophobic epidermis layers of onion and incubated for 48 h in the dark and moist chamber at 22°C (Viefhues et al., 2014).

### Pathogenicity Assay

Three-day-old mycelial plugs with 5 mm diameter or 10-day-old conidial suspensions (1.0–1.5 × 10^5^ spores/ml) were inoculated on 4-week-old detached tomato leaves and grape. Inoculated plant materials were incubated in 16 h daylight humid chamber at 22°C. Results were recorded after 4 days and 7 days. The experiment was repeated at least three times.

### Yeast Two-Hybrid (Y2H) Assay

The Y2H assay was conducted according to the manufacturer’s standard instructions (Clontech). The cDNA of *BcBAG1* was cloned into pGBKT7 as the bait vector and the cDNAs of HSP70-type chaperones were cloned into pGADT7 as the prey constructs, respectively. The pGBKT7-*BcBAG1* and each prey vector were co-transformed into the AH109 yeast strain to evaluate interactions. The positive and negative controls were from the Kit (Clontech).

### Arabidopsis Complementation Assays

*Arabidopsis thaliana* Col-0 and *atbag4* T-DNA knock out mutants were obtained from *Arabidopsis* Stock Center^4^. *Atbag4* homozygous mutants (SALK_027577C) were confirmed by PCR. The *BcBAG1* full-length cDNA was cloned into pCB302ES containing the 35S promoter and the HA-epitope tag (Hwang and Sheen, 2002). This construct was transferred into the *atbag4* knockout mutants by the floral dipping method (Zhang et al., 2006).

For salt stress assays, seeds were surface sterilized in 70% ethanol for 10 min and in 5% bleach solution for 5 min, and germinated on 1/2 Murashige and Skoog (MS) medium (Invitrogen) at 23°C for 5 days. The seedlings were transferred to fresh 1/2 MS medium containing 100 mM NaCl and grown at 23°C for 2 weeks to 5 weeks.

## Results

### Identification and Characterization of *BcBAG1*

To identify BAG proteins in *B. cinerea*, we searched *B. cinerea* B05.10 genome database with “BAG” as a query, and obtained one hit (Bcin10g01250.1/BC1G_05107). Additionally, based on Pfam and SMART programs, we search for all gene with BAG domain (BD domain) in genome database. Results indicated that there is only one gene with a single copy (Bcin10g01250.1/BC1G_05107) containing BAG domain, designated BcBAG1 hereafter. Phylogenetic analysis revealed that BcBAG1 shares low similarity with BAGs in yeast, plants and animals while it is closely related to BAG homologs from other filamentous fungi, e.g., *Sclerotinia sclerotiorum* (86.53%), *Magnaporthe oryzae* (57.04%), *Fusarium oxysporum* (52.01%), and *Aspergillus nidulans* (45.30%) (Supplementary Figure S1A and Supplementary Table S3). BcBAG1 contains a ubiquitin-like domain (UBL) at the N-terminus and a BAG domain at the C-terminus, encoding a 35 kD protein with 298 amino acids (Supplementary Figure S1B). The alignment also showed that most of the key interaction residues for BAG-HSC70/HSP70 binding are conserved in BAG proteins across filamentous fungi, yeast, Arabidopsis, and human (Supplementary Figure S1C).

To address the function of *BcBAG1*, we generated knockout mutants of *BcBAG1* (Δ*Bcbag1*), in the wild-type (WT) strain B05.10 (Supplementary Figure S2A). Two individual Δ*Bcbag1* lines, K3-7 and K8-6, were validated and selected for later use (Supplementary Figures S2B,C). We obtained three complemented strains by transforming the full-length *BcBAG1* under its native promoter into mutants and all strains equally restored the defects of Δ*Bcbag1*. Thus one complemented strain (*BcBAG1-Com*) was used in the following studies.

### BcBAG1 Is Required for Vegetative Growth, Conidiation, and Sclerotial Formation

**FIGURE 1:**
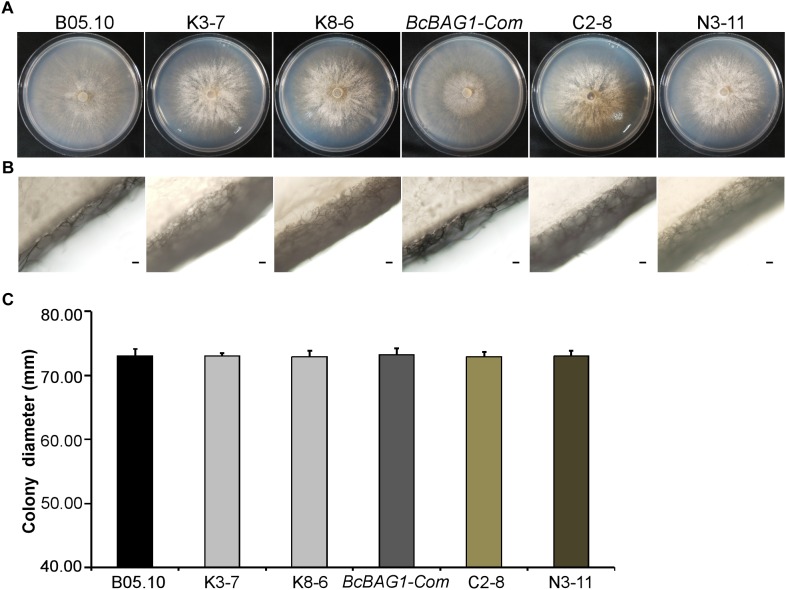
BcBAG1 is required for normal mycelial growth. Colonies **(A)** and aerial hyphae **(B)** of all strains were photographed after 3 days growth on PDA medium at 22°C. Scale bar: 100 μm. **(C)** Statistical analysis of the colony diameters of above strains. B05.10: the wild-type strain; K-3 and K8-6: Δ*Bcbag1* mutant lines; *BcBAG1-Com* (full-length *BcBAG1*), C2-8 (C-terminus of *BcBAG1*) and N3-11 (N-terminus of *BcBAG1*): complemented strains. Error bars represent the standard deviations from three independent experiments.

To evaluate the role of BcBAG1 during vegetative growth of *B. cinerea*, we examined hyphal growth on PDA. As shown in Figure 1A,C, the diameters of the Δ*Bcbag1* colonies were similar to WT strain B05.10 and the complementation strain *BcBAG1-Com*. However, Δ*Bcbag1* formed a thick hyphal layer on the surface of plates and the amount of aerial hypha drastically increased in comparison with B05.10 and *BcBAG1-Com* (Figure 1B). These results indicate that BcBAG1 influences on vegetative growth of *B. cinerea*.

**FIGURE 2:**
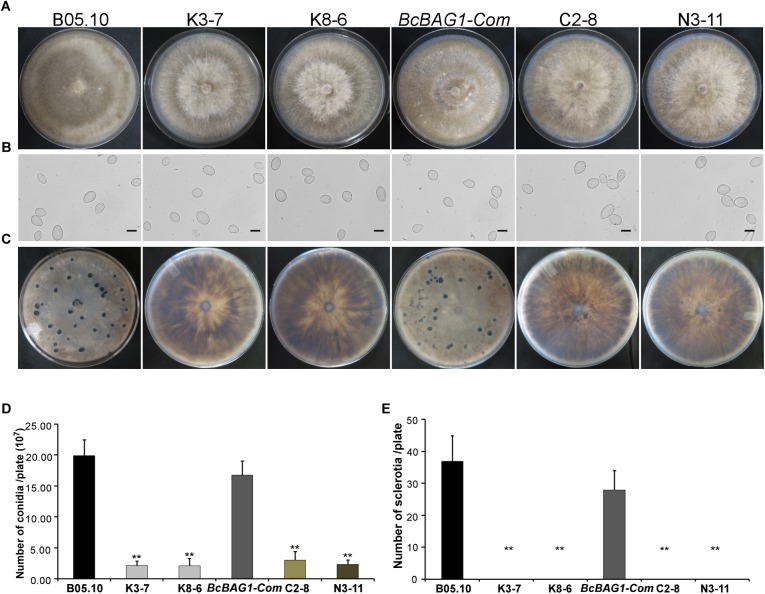
BcBAG1 is essential to conidiation and sclerotial formation. **(A)** Conidiation is affected by *BcBAG1* disruption. Strains grown on PDA for 10 days were examined by light microscopy. **(B)** Conidia shape comparison. Conidia were collected from 10 days colonies of strains on PDA. Scale bar: 10 μm. **(C)** Sclerotial formation is abolished in the Δ*Bcbag1* mutants. Strains were incubated PDA for 10 days in the dark. **(D)** Quantification of conidia production on PDA plates. **(E)** Quantification of sclerotia formation on PDA plates. Wild-type strain (B05.10), Δ*Bcbag1* mutant lines (K3-7 and K8-6), and complemented strains (*BcBAG1-Com*, C2-8 and N3-11). Error bars represent the standard deviations from three independent experiments and asterisks denote statistical significance (*P* < 0.01).

Given that wind-dispersal of conidia determines the severity of gray mold disease in the field (Leroch et al., 2013), we assessed the role of BcBAG1 in conidial production. Conidiation of B05.10, Δ*Bcbag1* and *BcBAG1-Com* from 10-day-old PDA culture was measured using microscopic examination. Although Δ*Bcbag1* still produced aerial mycelia (Figure 1B), conidiogenesis of Δ*Bcbag1* is significantly reduced (Figure 2A), in detail, the mutants produced approximately 2.1 × 10^7^ conidia/PDA plate, while the WT produced approximately 2.0 × 10^8^ conidia/PDA plate (*P* < 0.01) (Figure 2D). However, the conidia exhibited normal morphology between Δ*Bcbag1* and WT (Figure 2B). These results indicate that BcBAG1 involved in conidial production but do not affect conidial morphology in *B. cinerea*.

Melanization of sclerotia is considered as a survival strategy of various fungi when encountering harsh environments like over-wintering (Williamson et al., 2007). Here, we examined sclerotial formation following *BcBAG1* deletion in *B. cinerea*. After 4-week incubation on PDA in the dark, we observed that Δ*Bcbag1* mutants were unable to produce sclerotia, while B05.10 and *BcBAG1-Com* produced abundant sclerotia on PDA (Figure 2C,E), suggesting BcBAG1 is essential for sclerotial formation. Taken together, we reasoned that BcBAG1 plays a crucial role in regulating vegetative growth, conidiation and sclerotial formation in *B. cinerea*.

### BcBAG1 Is Involved in the Regulation of Hyphal Melanization

**FIGURE 3:**
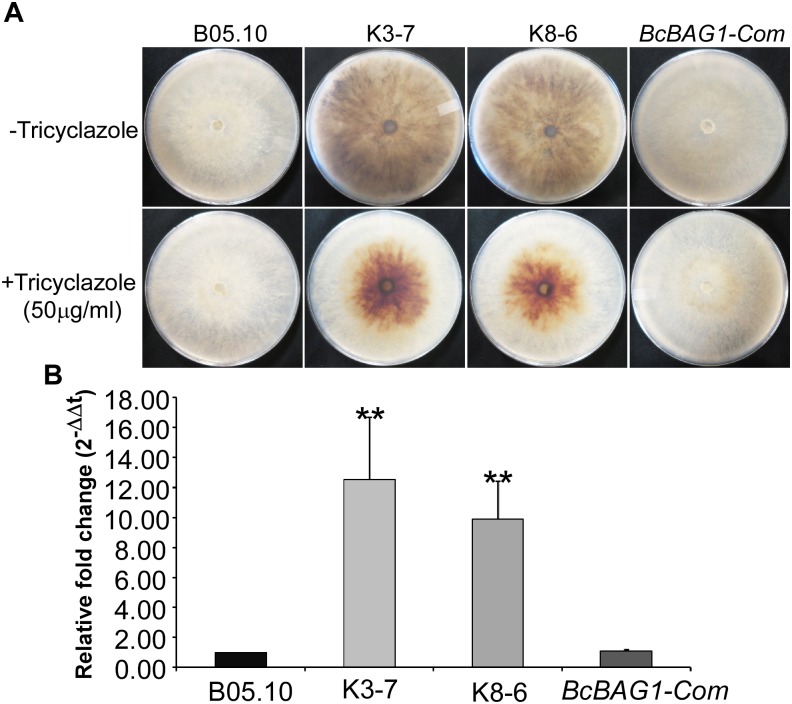
BcBAG1 negatively regulates hypal melanization. **(A)** Mycelial pigmentation were monitored on PDA for 7 days in darkness without (upper panel) and with (lower panel) tricyclazole (an inhibitor of DHN-melanin biosynthesis) in the wild-type (B05.10), Δ*Bcbag1* mutants (K-3 and K8-6) and complemented strain (*BcBAG1-Com*). **(B)** Transcription analysis of *THR1* (1,3,8-trihydroxynaphthalene reductase gene) by RT-PCR. Total RNAs of corresponding strains grown on PDB medium were extracted and conducted for RT-PCR, asterisks denote statistical significance (*P* < 0.01).

After incubating on PDA for 7 days, we noticed that Δ*Bcbag1* displayed increased generation of black pigment when compared to WT (Figure 3A). It has been reported that the dark pigmentation in fungi is due to the accumulation of 1,8 dihydroxynaphthalene (DHN) melanin (Henson et al., 1999). Melanin is a dark durable pigment that protects fungi against diverse environmental stresses, such as UV irradiation and temperature extremes (Bell and Wheeler, 1986; Butler and Day, 1998). We therefore examined whether BcBAG1 participates in melanin biosynthesis. The Δ*Bcbag1*, *BcBAG1-Com* and the WT strains were cultured on PDA supplemented with 50 μg/ml tricyclazole, a fungicide that specifically inhibits DHN-melanin biosynthesis (Thompson et al., 2000). The result showed tricyclazole was able to repress the massive melanin synthesis in Δ*Bcbag1* mutants caused by *BcBAG1* deletion (Figure 3A). Additionally, we instituted RT-PCR assays to monitor the expression level of *THR1* (1,3,8-trihydroxynaphthalene reductase gene), a key component in melanin biosynthesis pathway (Perpetua et al., 1996). Corresponding results obtained from RT-PCR analysis revealed a significantly up-regulation (about 10-folds) in the expression pattern of *THR1* in Δ*Bcbag1* compared to WT (Figure 3B). These data indicate that BcBAG1 negatively regulates melanin biosynthesis pathway to suppress melanin production in *B. cinerea*.

### BcBAG1 Is Required for Virulence of *B. cinerea*

**FIGURE 4:**
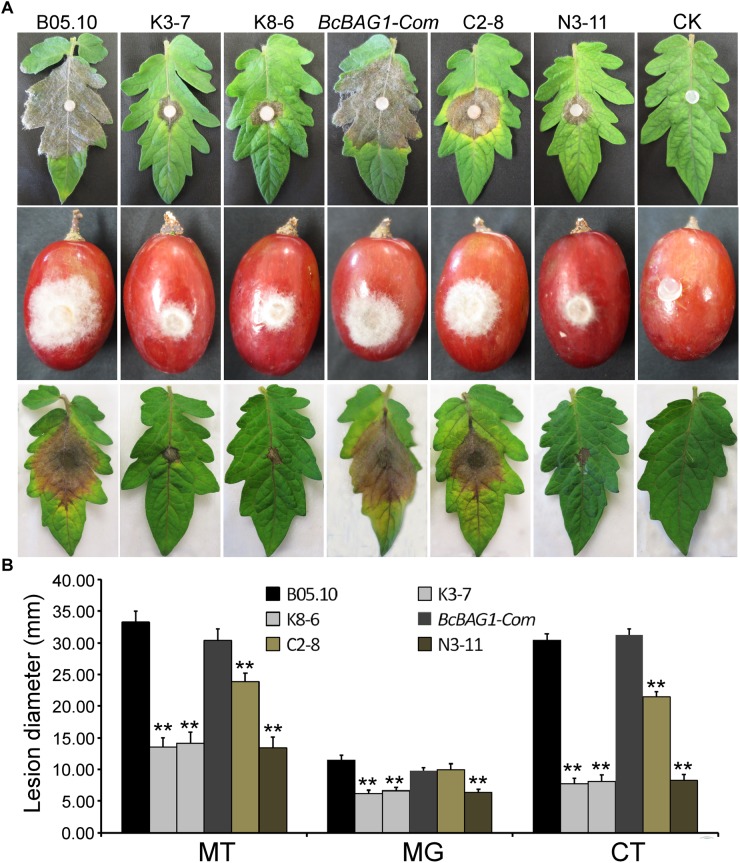
*BcBAG1* deletion mutants attenuated pathogenicity. **(A)** Inoculation assays were implemented on different host plants. Mycelial plugs from PDA after 3 days growth were inoculated on detached tomato leaves (Moneymaker) (upper panel) and wounded grapes (middle panel). The disease phenotype was recorded 4 days post inoculation. Conidial suspensions (1.0–1.5 × 10^5^ spores/ml) were inoculated on detached tomato leaves (Moneymaker) (lower panel) and the disease symptom was recorded 7 days post inoculation. **(B)** Quantification of lesion diameters of above inoculation. MT, mycelial plugs were inoculated on tomato leaves; MG, mycelial plugs were inoculated on grapes; CT, conidial suspensions were inoculated on tomato leaves. Wild-type strain (B05.10), Δ*Bcbag1* mutant lines (K3-7 and K8-6), and complemented strains (*BcBAG1-Com*, C2-8 and N3-11); CK, negative control. Error bars represent the standard deviations from three independent experiments and asterisks indicate statistical significance (*P* < 0.01).

To determine the role of BcBAG1 in pathogenicity and virulence of *B. cinerea*, we conducted infection assays by inoculating mycelial plugs containing the WT, Δ*Bcbag1* and *BcBAG1-Com*, on detached tomato leaves and grapes, respectively. Four days post-inoculation (dpi), Δ*Bcbag1* only initiated a small localized lesions, whereas the WT and the complemented strains have produced fully expanded lesions that were already at the soft rot stage (Figure 4A). We also performed infection assays on tomato leaves, with conidial suspensions (1.0 × 10^5^ spores/ml). Consistently, Δ*Bcbag1* showed apparently attenuated virulence compared to the WT and *BcBAG1-Com* (Figure 4A,B). These results indicate that BcBAG1 functions as a virulence factor by enhancing colonization on the hosts.

**FIGURE 5:**
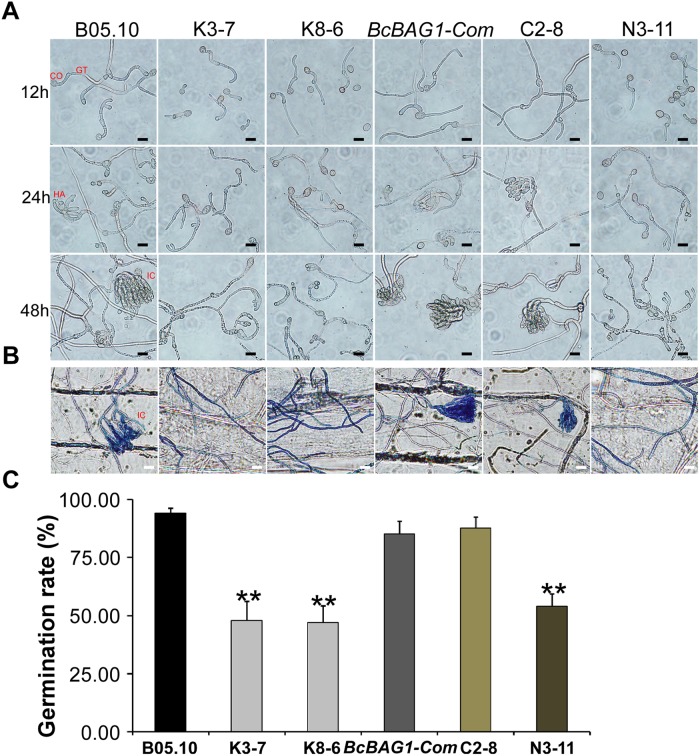
BcBAG1 is required for conidial germination and penetration. **(A)** Time course of conidial germination on glass slides when conidia were suspended in 10 mM KH_2_PO_4_ and glucose solution (pH 6.0). CO, conidia; GT, germ tubes; HA, appressoria; IC, infection cushions. Scale bar: 20 μm. **(B)** Penetration assay on onion epidermal cells. Onion epidermal cells were inoculated with conidial suspension (5.0 × 10^4^ spores/ml) and the penetration was observed 48 h post incubation (hpi). External fungal structures are stained blue with cotton blue, whereas penetrated hyphae remain unstained. Scale bar: 20 μm. **(C)** Quantification of conidial germination rates after 12 hpi on hydrophobic glass slide. Error bars represent the standard deviations from three independent experiments (more than 100 conidia per strain). Asterisks denote statistical significance (*P* < 0.01).

During infection, *B. cinerea* produces three types of penetration structures, including germ tube apices (GA), appressoria (HA) and infection cushions (IC) (Vandenheuvel and Waterreus, 1983). To investigate whether the weak virulence of Δ*Bcbag1* resulted from penetration defects, we evaluated conidial germination using hydrophobic coverslips and the penetration on onion epidermis cells for microscopic observation (Figure 5A). Although all strains initiated germination 12 h post-incubation (hpi), the germination rate of Δ*Bcbag1* was significantly reduced (by 50%) compared to the germination recorded for the WT and *BcBAG1-Com* (Figure 5C). Moreover, deletion of *BcBAG1* delayed the onset of appressoria formation at 24 hpi and the development of infection cushions (IC) at 48 hpi (Figure 5A). Cotton blue was used to stain the appressorium-like structure, the infection cushion and the hyphae of *B. cinerea* on onion epidermal cells. As shown in Figure 5B, the conidia of WT and *BcBAG1-Com* germinated, and the infection cushions surrounded by abundant invasive hyphae were typically developed for penetration on the onion epidermis 48 hpi. In contrast, Δ*Bcbag1* attenuated the ability to form infection cushions and only few germ tubes and invasive hyphae appeared on onion cells. These results suggest that the reduced virulence of Δ*Bcbag1* is correlated with the developmental defects of infection and penetration structures.

### Both UBL and BAG Domains Contribute to BcBAG1 Function

BcBAG1 possesses an N-terminal ubiquitin-like (UBL) domain and a conserved C-terminal BAG domain (BD) (Supplementary Figure S1B). Human BAG-1 interacts with HSP70 via its BAG domain and utilizes the UBL domain in targeting the chaperone cofactor to the 26S proteasome for degradation (Demand et al., 2001). The UBL/BAG domain proteins in *S. pombe*, SpBAG101 and SpBAG102, display similar interaction pattern to human BAG-1 (Kriegenburg et al., 2014; Poulsen et al., 2017). To validate the functionality of the UBL and the BAG domain of BcBAG1, we generated two truncated forms of BcBAG1; BcBAG1A^1-141^ and BcBAG1B^142-298^ (Supplementary Figure S1B), containing UBL and BAG domain, respectively. The BcBAG1A^1-141^ and BcBAG1B^142-298^ constructs were subsequently transformed into Δ*Bcbag1* for complementation, designated as N3-11 and C2-8, respectively. Both N3-11 and C2-8 could not effectively rescue defects in conidia production and sclerotia formation (Figure 2). Interestingly, C2-8 could partially restored conidial germination, infection structure formation and pathogenicity to WT levels (Figure 4, 5). These data suggest that both UBL and BAG domains are necessary for integral BcBAG1 function and we further inferred that the BAG domain plays an indispensable role for pathogenicity of *B. cinerea*.

### BcBAG1 Modulates Multiple Stress Responses in *B. cinerea*

**FIGURE 6:**
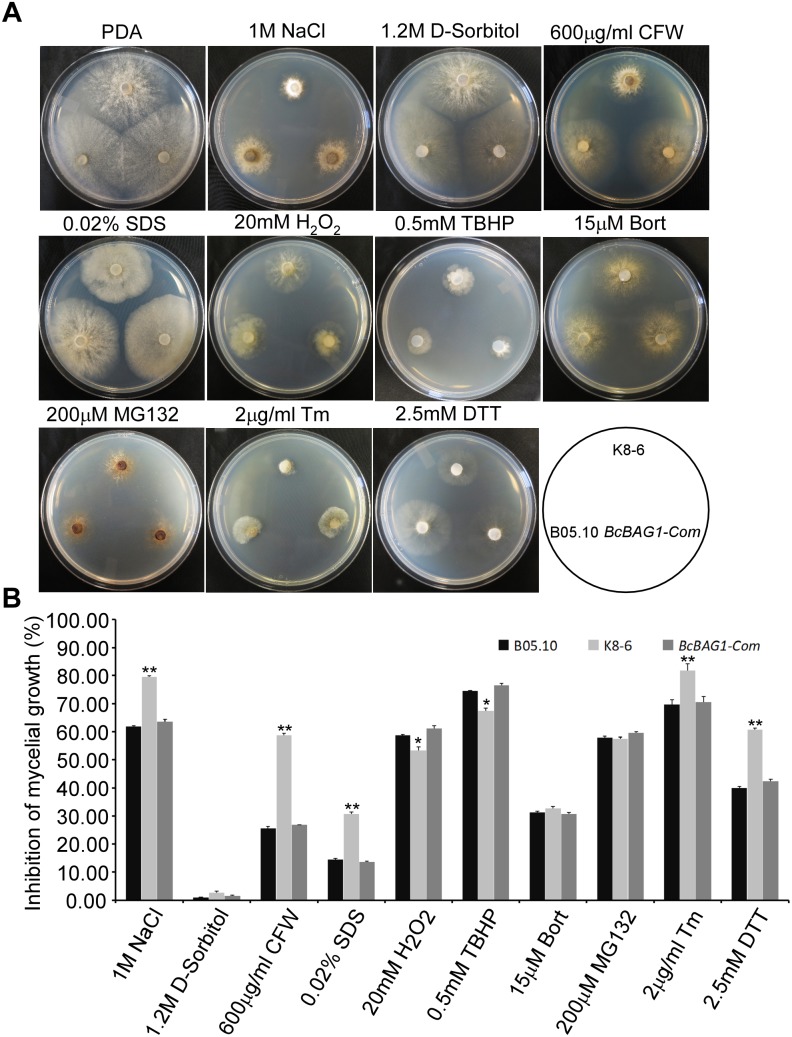
BcBAG1 responses to various stress conditions. **(A)** Wild-type B05.10, Δ*Bcbag1* (K8-6) and *BcBAG1-Com* strains were cultured on PDA medium supplemented with various stresses inducers and photographed after 3 days. **(B)** Statistical analyses of the inhibition rates of all strains under different stresses. Error bars indicate the standard deviations from three independent experiments and asterisks indicate statistical significance (*P* < 0.05; *P* < 0.01).

BAG family members are involved in cell protection during variable biotic and abiotic stress responses (Doukhanina et al., 2006; Behl, 2016). To investigate the function of BcBAG1 in response to environmental stress, we examined the sensitivity of Δ*Bcbag1* to various abiotic stress stimuli. As shown in Figure 6, Δ*Bcbag1* was more sensitive to salt stress (1 M NaCl) than WT, but no different with WT to another osmotic stress inducer 1.2 M D-sorbitol. Mycelial growth in response to cell wall stress inducers (0.6 mg/ml CFW; and 0.02% SDS) was measured. Δ*Bcbag1* showed dramatically increased sensitivity to both CFW and SDS compared to WT (Figure 6). In contrast, when exposing to oxidative stimuli, 20 mM H_2_O_2_ and 0.5 mM TBHP, Δ*Bcbag1* led to an average ∼7% (20 mM H_2_O_2_) and ∼8% (0.5 mM TBHP) lower growth inhibition rate than the WT strain (Figure 6), indicating that disruption of *BcBAG1* is more resistance to oxidative stress.

Human BAG-1 is a coupling factor between HSP70 and 26S proteasome (Luders et al., 2000). BAG3, as a co-chaperone, forms a complex with HSP70 to facilitate the degradation of ubiquitinated proteins via the proteasome or autophagy pathways (Gamerdinger et al., 2011; Minoia et al., 2014). Thus, we examined the role of BcBAG1 in proteasome degradation by testing the sensitivity of Δ*Bcbag1* to proteasome inhibitors, MG132 and Bort (Huang and Chen, 2009). Unexpectedly, results showed that the growth rate of Δ*Bcbag1* is not statistically different from WT and *BcBAG1-Com* (Figure 6), implying that BcBAG1 is not involved in response to proteasome inhibitors, MG132 and Bort. Although human BAG3 mediate the responses to Bort and MG132 (Judge et al., 2017), BcBAG1 does not share the same role in this perspective as the human counterpart.

### BcBAG1 Negatively Regulates Unfolded Protein Response (UPR)

**FIGURE 7:**
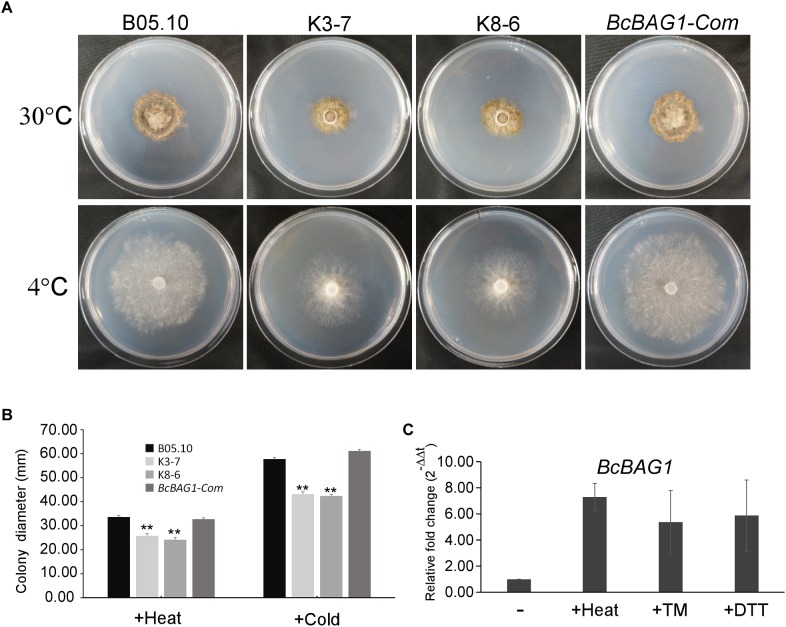
*BcBAG1* knock-out mutants are susceptible to ER stress conditions. **(A)** Wild-type B05.10, Δ*Bcbag1* (K3-7 and K8-6) and *BcBAG1-Com* strains were grown on PDA medium at 30°C and 4°C, respectively, for 7 days. **(B)** Statistical analysis of the colony diameters of above strains. **(C)** The expression of *BcBAG1* is induced by heat, Tm, and DTT treatments. Quantitative RT-PCR was used to evaluate the *BcBAG1* transcript levels in the wild-type B05.10 following heat treatment (50°C for 30 min), tunicamycin (Tm, 15 μg/ml for 1 h) and DTT (20 mM for 1 h). Error bars represent the standard deviations from three independent experiments. Asterisks denote statistical significance (*P* < 0.01).

Previous work revealed that Arabidopsis BAG7 (AtBAG7) functions as an ER stress co-chaperone to maintain the UPR and protect plants from ER stress-induced cell death (Williams et al., 2010). Except AtBAG4, BcBAG1 shares relative higher identity to AtBAG7 in comparison to other Arabidopsis BAGs (Supplementary Table S3), we therefore examined whether BcBAG1 plays a role in ER stress signaling pathway. ER stress can be induced by chemical compounds, e.g., Tm or DTT (Oslowski and Urano, 2011). Besides, environmental/abiotic stress including excessive heat and cold also trigger ER stress (Williams et al., 2010). Accordingly, we cultured the WT, Δ*Bcbag1* and *BcBAG1-Com* on media supplemented with 2 μg/ml Tm and 2.5 mM DTT for 3 days at 22°C. The results showed that the growth of Δ*Bcbag1* was strongly inhibited by Tm and DTT with a much higher inhibition rate comparing to WT and *BcBAG1-Com* (Figure 6). In addition, after incubating on PDA under heat (30°C) and cold (4°C) conditions for 7 days, Δ*Bcbag1* was more sensitive than the WT and *BcBAG1-Com* to both heat and cold treatments (Figure 7A–C). Quantitative real-time (qRT-PCR) analysis demonstrated that the transcription of *BcBAG1* was highly induced upon above ER stress conditions, including heat treatment (50°C for 30 min), Tm (2 μg/ml for 1 h), or DTT (20 mM for 1 h) (Figure 7C), indicating that *BcBAG1* is responsible for ER stress tolerance.

**FIGURE 8:**
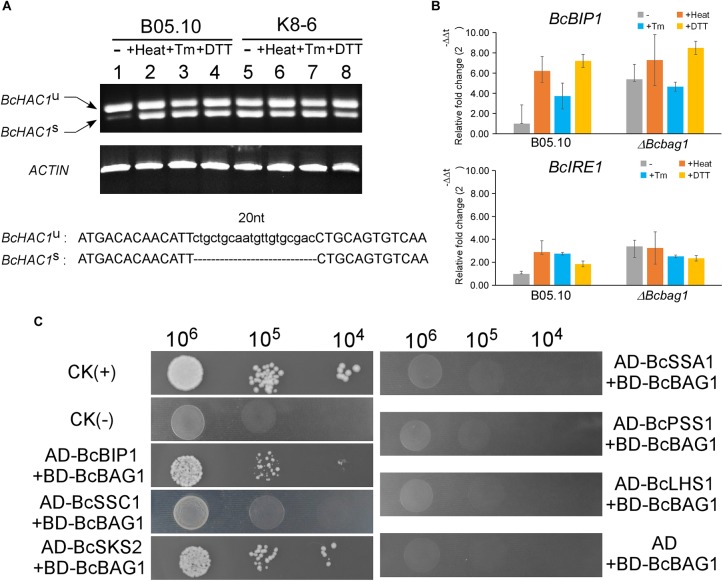
BcBAG1 involves in the activation of unfolded protein response (UPR) and interacts with two HSP70-type chaperones. **(A)** BcBAG1 negatively regulates the splicing of *BcHAC1* mRNA under normal condition. RT-PCR was performed using total RNAs extracted from the wild-type B05.10 and Δ*Bcbag1* mutant K8-6 grown in liquid PDB for 2 days (lane 1 and lane 5), respectively. The rest of total RNAs were collected from the strains grown in liquid PDB for 2 days then treat with ER stresses. Lane 2 and lane 6: B05.10 and K8-6 subjected to 50°C for 30 min, respectively; lane 3 and lane 7 treated with 15 μg/ml Tm for 1 h, respectively; lane 4 and lane 8: B05.10 and K8-6 treated with 20 mM DTT for 1 h, respectively. The *ACTIN* gene was used as the internal control. DNA sequence alignment of the *BcHAC1^U^* and *BcHAC1^S^*. The 20 nt atypical intron is indicated on the bottom panel. **(B)** The expression profiles of UPR correlated genes, *BcBIP1* and *BcIRE1* during ER stress by real-time PCR. Total RNAs of corresponding strains grown in PDB medium or after stress treatments were extracted and conducted for real-time PCR. Error bars represent the standard deviations from three independent experiments. **(C)** Yeast two-hybrid assay between BcBAG1 and HSP70 family members. The yeast transformants diluted to specified concentrations (cell/ml) were plated onto SD/-Leu/-Trp/-His. The interaction of pGBKT7-53 with pGADT7-T was used as the positive control CK (+) and the interaction of pGBKT7-Lam with pGADT7-T as the negative control CK (–), respectively.

To cope with ER stress, eukaryotes utilize UPR to alleviate the detrimental effects (Schroder and Kaufman, 2005). In *S. cerevisiae*, UPR is sensed by transmembrane protein kinase and ribonuclease (RNase) IRE1 and initiated with IRE1-mediated splicing of an unconventional intron (252-nucleotide) from the *HAC1* transcript (Cox and Walter, 1996; Ruegsegger et al., 2001). HAC1 encodes a basic leucine zipper-type (bZIP) transcription factor, and the splicing of *HAC1* regulates the expression of UPR target genes thus to mitigate ER stress (Mori et al., 1996). To explore the underlying mechanism within the sensitivity of Δ*Bcbag1* to ER stress, we detected the presence of the spliced and unspliced form of *BcHAC1* mRNA by RT-PCR. Spliced form of *BcHAC1* (*BcHAC1^S^*) in B05.10 was significantly increased upon ER stress, suggesting *B. cinerea* shares the similar *HAC1* splicing process with other organisms during UPR (Figure 8A). Notably, under normal conditions, the amount of *BcHAC1^S^* was drastically more abundant in Δ*Bcbag1* than the WT (Figure 8A, upper panel). More interestingly, *BcHAC1* was constitutively spliced in Δ*Bcbag1* under both conditions that are with/with-out stress (Figure 8A). DNA sequencing confirmed that a 20 nucleotide of the fragment was absent in the spliced form compared to the unspliced form (Figure 8A, lower panel). Meanwhile, we examined the expression of UPR-related genes in *B. cinerea*, BcBIP1 and BcIRE1, homologs of ER chaperone KAR2/BIP1 and ER stress sensor/transducer IRE1 in *S. cerevisiae*, respectively (Pincus et al., 2010). Both *BcBIP1* and *BcIRE1* were induced following ER stress in the WT (Figure 8B). Without stress, the expression levels of *BcIRE1* and *BcBIP1* were increased by threefold and fivefold, respectively, in Δ*Bcbag1* (Figure 8B). All these results indicates that, *BcBAG1* deletion causes constitutive activation of UPR through negatively regulating the expression of *BcBIP1*, *BcIRE1* and the splicing of *BcHAC1* mRNA. We speculate that BcBAG1 effectively repress the harmful and excessive constitutive activation of UPR, thus to maintain the proper UPR level during ER stress signaling pathway.

### BcBAG1 Binds HSP70-Type Chaperones

The heat shock proteins HSP70 family play crucial roles in assisting a variety of protein folding processes (Mayer and Bukau, 2005). Human BAG-1 binds the ATPase domain of Hsc70 to stimulates Hsc70 ATP hydrolysis which results in the release of ADP from Hsc70, thereby regulates specific protein folding and maturation pathways (Hohfeld and Jentsch, 1997). To ascertain whether BcBAG1 bind HSP70-type chaperone in *B. cinerea*, we performed the yeast two-hybrid assay to establish possible interaction between BcBAG1 and the HSP70 family members, including BcBIP1, BcSSC1, BcSKS2, BcSSA1, BcPSS1, and BcLHS1, which are homologs of HSP70 family in the fission yeast. Results showed that BcBAG1 only interacted with BcBIP1 and BcSKS2 (Figure 8C), demonstrating BcBAG1 does function as a co-chaperone of HSP70 proteins, in accordance with that has been reported in other systems (Gassler et al., 2001; Sondermann et al., 2002; Poulsen et al., 2017).

### BcBAG1 Restores Salt Stress Tolerance to *atbag4*

**FIGURE 9:**
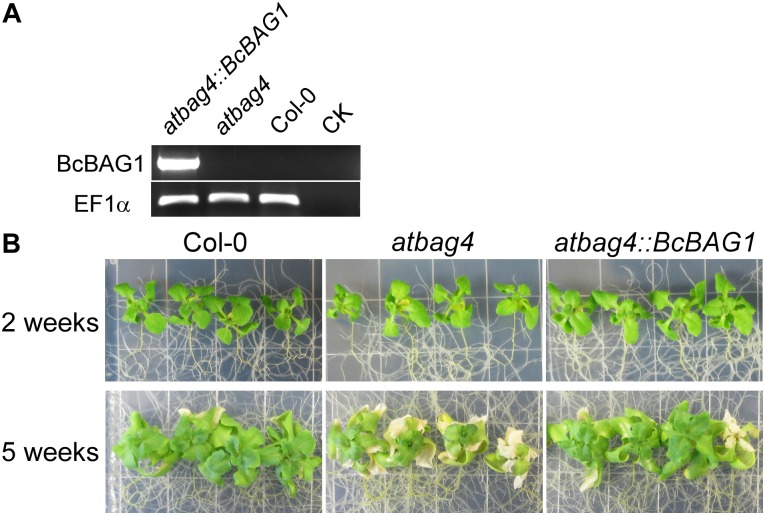
BcBAG1 restores the salt stress tolerance of AtBAG4 T-DNA knock-out mutant. **(A)** BcBAG1 ectopically expressed in Arabidopsis plants. BcBAG1 driven by 35S promoter was transformed in to *atbag4* mutant (*atbag4::BcBAG1*) and the expression of BcBAG1 was confirmed by RT-PCR. Taking *atbag4* and the wild-type plant Col-0 as controls. CK, negative control. The *EF1a* gene was used as the internal control. **(B)** Col-0, *atbag4* and *atbag4::BcBAG1* were subjected to salt stress. Plants were grown on half-strength Murashige and Skoog (MS) medium with 2% sucrose and subjected to 100 mM NaCl treatment. Plants growth was recorded after 2 weeks and 5 weeks.

Using NCBI blastp and blastn programs, we found BcBAG1 is predicted to be the closest homolog of Arabidopsis BAG protein AtBAG4, in light of the amino acid sequences of the BAG domains between BcBAG1 and AtBAG4 share 33% identity and 50% similarity (Supplementary Figures S1A,C and Supplementary Table S3). To determine whether BcBAG1 is able to functionally complement the AtBAG4 T-DNA mutants, full-length *BcBAG1* was overexpressed using the cauliflower mosaic virus 35S promoter in the *atbag4* T-DNA mutants (*atbag4::BcBAG1*). Expression levels of *BcBAG1* in *atbag4* was confirmed by RT-PCR (Figure 9A). Previous studies indicated that *atbag4* mutants were more susceptible to salt stress (100 mM NaCl) compared to the WT Col-0 (Doukhanina et al., 2006). Here, we performed the salt tolerance assay for *atbag4::BcBAG1*, taking Col-0 and *atbag4* as the positive and negative controls, respectively. Arabidopsis seedlings were cultured on 1/2 MS medium supplemented with 100 mM NaCl. After 2 weeks treatment, no difference was observed from seedlings. However, 5 weeks later, *atbag4* mutants displayed massive chlorosis and bleaching of leaves, while Col-0 and *atbag4::BcBAG1* plants grew well and exhibited nearly the same growth tendency under salt conditions (Figure 9B). These observations demonstrate that BcBAG1 can be ectopically expressed in Arabidopsis and fully restore salt tolerance in *atbag4*.

## Discussion

As an evolutionarily conserved group, BAG proteins from yeast to plants and mammals have been associated with regulation of PCD and cell protection. Recently, they have also been found to play an important role in autophagy, UPR and ubiquitin-proteasome system (Kabbage et al., 2017). However, the identification and characterization of BAG proteins in phytopathogenic fungi is rare. In this study, we explored the *B. cinerea* genome and identified a unique *BAG* gene, *BcBAG1*. Targeted deletion of *BcBAG1* exerted strong adverse effects on vegetative growth, conidiation, sclerotia formation, hyphal melanization, stress response, conidial germination, penetration and virulence, suggesting that the pleiotropic function of BAGs delineated in mammals and plants appears to be maintained in *B. cinerea*. The BAG domain of BcBAG1 shares highest similarity with AtBAG4 in Arabidopsis, also an ectopic expression of BcBAG1 fully restored salt tolerance of *atbag4* mutants (Figure 9). In addition, deletion of *BcBAG1* resulted in increased sensitivity to salt, cell wall stressors, ER stress inducers, and heat or cold treatments. These results parallel plant AtBAG4 studies in which AtBAG4-overexpressing transgenic tobacco plants confer tolerance to a wide range of stresses such as UV light, cold, salt treatments (Doukhanina et al., 2006). From this perspective, the cytoprotective function of BAGs in response to diverse stresses is relatively conserved between the fungi and plants.

Our previous studies have addressed the importance of AtBAG7 in the maintenance of the UPR and the mechanisms of ER-localized co-chaperone AtBAG7 in stress protection (Williams et al., 2010; Li et al., 2017). Interestingly, BcBAG1 showed a close evolutionary relationship with AtBAG7 among plant BAGs (Supplementary Figure S1A and Supplementary Table S3). Moreover, Δ*Bcbag1* were more sensitive to ER stress stimuli (Figure 6) and expression of *BcBAG1* was induced under ER stress (Figure 7B), suggesting that both BcBAG1 and AtBAG7 are functionally associated with the ER stress. Of note, we found that the deletion of *BcBAG1* gives rise to the constitutive activation of UPR with high levels even without stress (Figure 8A,B), indicating that BcBAG1 is necessary for the inhibition of excess UPR under normal condition. Given the defects in fungal development and differentiation caused by deletion of *BcBAG1*, we speculate that under normal conditions, the abnormal activated UPR is actually harmful to the fungus. It has been reported that moderate activation of UPR is necessary for ER recovery when responding to stress in *S. cerevisiae* (Chawla et al., 2011). Thus, we suggest that constitutive activation of UPR in Δ*Bcbag1* results in loss of normal ER protein-folding capacity, which impinges upon the ability to sustain resistance to ER stress. Thus, Δ*Bcbag1* displayed growth defects during ER stress. In addition, as a multifaceted HSP70 molecular chaperone, BIP ensures an appropriate response to restore protein folding homeostasis to the ER by providing a buffer for inactive IRE1 (Pincus et al., 2010). Our result revealed that disruption of *BcBAG1* increases the expression of *BcBIP1* and *BcIRE1* (Figure 8B) and BcBAG1 interacts with BcBIP1 (Figure 8C). We conclude that the regulation of UPR by BcBAG1 correlates to BcBIP1. The relationship between BcBAG1/BcBIP1/BcIRE1 and how BcBAG1 modulates the ER machinery require further studies.

Previous studies of human BAG-1/HSC70 complex revealed that BAG-1 exploits Glu^212^, Asp^222^, Arg^237^, and Gln^245^ residues in the BAG domain to bind with Hsc70 ATPase domain (Sondermann et al., 2001). Multiple alignment showed that these residues, with the exception of Glu^212^, are highly conserved in BcBAG1 BD (Supplementary Figure S1C), implying that BcBAG1 interacts with HSP70 in a similar manner as with human BAG-1. We did examine the interaction between BcBAG1 and HSP70 proteins by Y2H and found that BcBAG1 exclusively interacts with BcBIP1 and BcSKS2 in the HSP70 family, but fails to bind to BcSSC1, BcSSA1, BcPSS1, and BcLHS1 (Figure 8C). Previous studies demonstrated that the *S. cerevisiae* BAG protein SNL1 interacts with HSP70 family members including SSA1, SSB1, SSB2, SEE1, and SEE2 (Sondermann et al., 2002; Verghese and Morano, 2012; Abrams et al., 2014). While the *S. pombe* BAG proteins SpBAG101 and SpBAG102 exclusively interact with SSA1, SSA2, and SKS2 (Poulsen et al., 2017). These data suggest BAGs from different origins show different affinity and specificity to HSP70s members. Notably, the interaction between BcBAG1 and BcSKS2 is conserved from that in yeast studies. BcSKS2 is the homolog of fission yeast ribosome-associated chaperone SKS2, and the *S. cerevisiae* orthologs of fission yeast SKS2, called SSB1 and SSB2. SSB1/2 chaperones play a dual role in *de novo* protein folding and ribosome biogenesis (Mudholkar et al., 2017). We conjectured that the binding to primary HSP70 chaperone might be responsible for the functional pleiotropy of BcBAG1.

Apart from the BAG domain, BcBAG1 also contains a UBL domain. Human BAG-1 function as a link between HSC/HSP70 and 26S proteasome degradation system via its UBL domain, chaperone-bound substrates are released and degraded (Luders et al., 2000; Alberti et al., 2002). Co-precipitation experiments in fission yeast provide direct evidence that both SpBAG101 and SpBAG102 interact with 26S proteasomes depend on the UBL domain (Poulsen et al., 2017). However, we found that response to proteasome inhibitors, e.g., Bort and MG132, by *BcBAG1* deletion was unaffected in fungus (Figure 6). Besides, the result of ubiquitination assay confirmed that deletion of *BcBAG1* does not alter the levels of ubiquitination (Supplementary Figure S3). Therefore, we inferred that BcBAG1 is not a key player in the proteasome degradation. However, we cannot exclude the possibility that other proteasome inhibitors may work on BcBAG1 or BcBAG1 can be targeting some proteasome substrate for degradation.

*Botrytis cinerea*, however, integrates a number of hurdles that must be traversed for successful colonization and defense against the plethora of plant hosts that are encountered (Elad et al., 2007). Therefore, it is relatively difficult to present a precise mechanism for the alteration of pathogenicity and virulence in the host. Based on our data, we supposed that the attenuated pathogenicity of Δ*Bcbag1* is directly or indirectly related to the following reasons. First, Δ*Bcbag1* showed a decrease of conidial germination, and delayed the formation of appressoria and infection cushions on an artificial surface and onion epidermis (Figure 6). At the same time, we observed that BcBAG1 resides in the cytoplasm throughout growth stages, but the localization is altered and most likely concentrated at the infection spots during invasion (Supplementary Figure S4), suggesting the expression of *BcBAG1* contributes to the formation of penetration structures. Therefore, the impairment of penetration structures in Δ*Bcbag1* weakens the ability of breaching the host tissues to effectively cause disease. Second, it is reported that cell wall integrity is crucial for *B. cinerea* virulence and pathogenicity (Soulie et al., 2003, 2006; Cui et al., 2013). Δ*Bcbag1* increased sensitivity to cell wall stressors, CFW and SDS (Figure 6), indicating BcBAG1 involves in cell wall integrity pathway. Therefore, defect in cell wall integrity is one key to the reduction of virulence. Third, melanin is a factor affecting the virulence of *B. cinerea*. BcPKS13 and BcBRN1, encoding polyketide synthase and tetrahydroxynaphthalene (THN) reductases, respectively, both are involving in fungal DHN melanin biosynthesis (Zhang et al., 2015). Loss of *BcPKS13* and *BcBRN1* blocks melanization resulting in enhanced virulence. Conversely, overexpression of *BcBRN1* enhances melanization, decreases secretion for virulence factors such as several hydrolytic enzymes and oxalic acid, and attenuated virulence (Zhang et al., 2015). From this perspective, an increment of melanin biosynthesis negatively affects the pathogenesis of *B. cinerea*. Consistently, our results showed that BcBAG1 negatively regulates melanin biosynthesis (Figure 3), thus the increased mycelial melanin biosynthesis suppress fungal virulence for Δ*Bcbag1*. Fourth, previous studies indicated UPR plays as a central regulator of fungal pathogenesis (Heimel et al., 2013; Guillemette et al., 2014; Krishnan and Askew, 2014a,b). Our study found that BcBAG1 is responsible for proper maintenance of UPR, as a result, abnormal activation of UPR in Δ*Bcbag1* could cause the attenuated virulence.

In summary, this paper details the biological functions of BcBAG1. We have shown that BcBAG1 exhibits functional versatility and is involved in fungal development, differentiation, stress response, and pathogenicity. BcBAG1 acts as a co-chaperone of HSP70 and is a key regulator required for maintenance of the UPR. In light of these findings, future studies involving translocation of BcBAG1 during infection and identification of other targets are of interest. Taken together, these results demonstrate the importance of the BAG family in filamentous fungus cell death pathways and cytoprotection.

## Author Contributions

HZ and MD designed the research. HZ performed the experiments. HZ, YL, and MD analyzed the data. HZ, YL, MD, and ZW wrote the article. MD and ZW revised and approved the manuscript.

## Conflict of Interest Statement

The authors declare that the research was conducted in the absence of any commercial or financial relationships that could be construed as a potential conflict of interest.

## Footnotes

1 http://fungi.ensembl.org/Botrytis_cinerea/Info/Index

2 https://pfam.xfam.org/

3 https://www.ebi.ac.uk/interpro/

4 https://www.arabidopsis.org/

## Supplementary Material

Figure S1 Phylogenetic and sequence analysis of BAG1 in *Botrytis cinerea* (BcBAG1). **(A)** The phylogenetic tree of BAG proteins. Evolutionary analyses were conducted in MEGA7.0. The evolutionary history was inferred by using a neighbor-joining method based on the amino acid sequences. The numbers at nodes inferred the percentage of their occurrence in 10,000 bootstrap replicates. Species names and GenBank accession numbers of each sequence are represented as follows: SsBAG1 (*Sclerotinia sclerotiorum*, XP_001591798.1); MoBAG1 (*Magnaporthe oryzae*, XP_003710309.1); FoBAG1 (*Fusarium oxysporum f. sp. Lycopersici*, XP_018239181.1); NcBAG1 (*Neurospora crassa*, XP_961586.1); AnBAG1 (*Aspergillus nidulans*, XP_661815.1); UmBAG1 (*Ustilago maydis*, KIS67500.1); SpBAG101 (*Schizosaccharomyces pombe*, NP_596760.1); SpBAG102 (*Schizosaccharomyces pombe*, NP_595316.1); ScSNL1 (*Saccharomyces cerevisiae*, KZV10602.1); HsBAG-1M (*Homo sapiens*, NP_001336215.1); HsBAG2 (*Homo sapiens*, NP_004273.1); HsBAG3 (*Homo sapiens*, NP_004272.2); HsBAG4 (*Homo sapiens*, NP_004865.1); HsBAG5 (*Homo sapiens*, NP_001015049.1); HsBAG6 (*Homo sapiens*, P46379.2); AtBAG1 (*Arabidopsis thaliana*, NP_200019.2); AtBAG2 (*Arabidopsis thaliana*, NP_568950.2); AtBAG3 (*Arabidopsis thaliana*, NP_196339.1); AtBAG4 (*Arabidopsis thaliana*, NP_190746.2); AtBAG5 (*Arabidopsis thaliana*, NP_172670.2); AtBAG6 (*Arabidopsis thaliana*, AEC10664.1); AtBAG7 (*Arabidopsis thaliana*, NP_201045.1). **(B)** Schematic diagram of BcBAG1. Purple red and green boxes indicate the UBL and BAG domain of BcBAG1, respectively. **(C)** Sequence alignment of the conserved BAG domain from different organisms. Three predicted helixes labeled on the top. Conserved residues which involving in binding of BAG protein to Hsc70 ATPase domain in human HsBAG-1M are indicated by red arrow, and residues critical to packing interactions are highlighted by blue arrow.

Figure S2 Sketch of gene deletion and identification of deletion mutants and complemented transformants. **(A)** Diagram of targeted gene replacement. **(B)** RT-PCR confirmation. Lane 1:100 bp DNA Ladder (NEB); lanes 2 and 3: K3-7 and K8-6, respectively; lane 4: B05.10; lanes 5 and 6: complemented transformants; lane 7: PCR negative control. **(C)** Southern blotting confirmation. Total genomic DNAs were digested by *PvuI*, and a DNA fragment in the upstream of 5′ terminus of BcBAG1 was selected and labeled as the probe shown in panel **(A)**. M: 1 kb DNA Ladder (NEB).

Figure S3 Disruption of *BcBAG1* does not affect protein ubiquitination. Total protein extracts from corresponding strains was western blotted with (upper panel) an anti-Ub antibody (P4D1) and stained with Coomassie brilliant blue (lower panel) as the loading control. B05.10: the wild-type strain; K8-6: Δ*Bcbag1* mutant lines; C2-8 (C-terminus of *BcBAG1*) and N3-11 (N-terminus of *BcBAG1*).

Figure S4 Subcellular localization of BcBAG1. GFP-BcBAG1 was overexpressed in the Δ*Bcbag1* mutant (OG2-7) and the fluorescence for BcBAG1 localization at different stages was visualized by confocal microscopy. **(A)** Vegetative hyphae; **(B)** conidia; **(C,D)** conidia on hydrophobic glass slides for 12 and 24 h, respectively. **(E,F)** Both are conidia on onion epidermal cells for 24 h and 48 h, respectively. Scale bars: 20 μm.

Table S1 Wild-type and mutant strains of *Botrytis cinerea* used in this study.

Table S2 Oligonucleotide primers used in this study.

Table S3 Similarity of full length and BAG domain amino acid sequence between BcBAG1 and other BAGs.
