# Supplementary figures and images for: Cytoprotective Co-chaperone BcBAG1 Is a Component for Fungal Development, Virulence, and Unfolded Protein Response (UPR) of Botrytis cinerea
Source: Front Microbiol. 2019 Apr 9;10:685. doi: 10.3389/fmicb.2019.00685 (PMC6467101; doi:10.3389/fmicb.2019.00685)

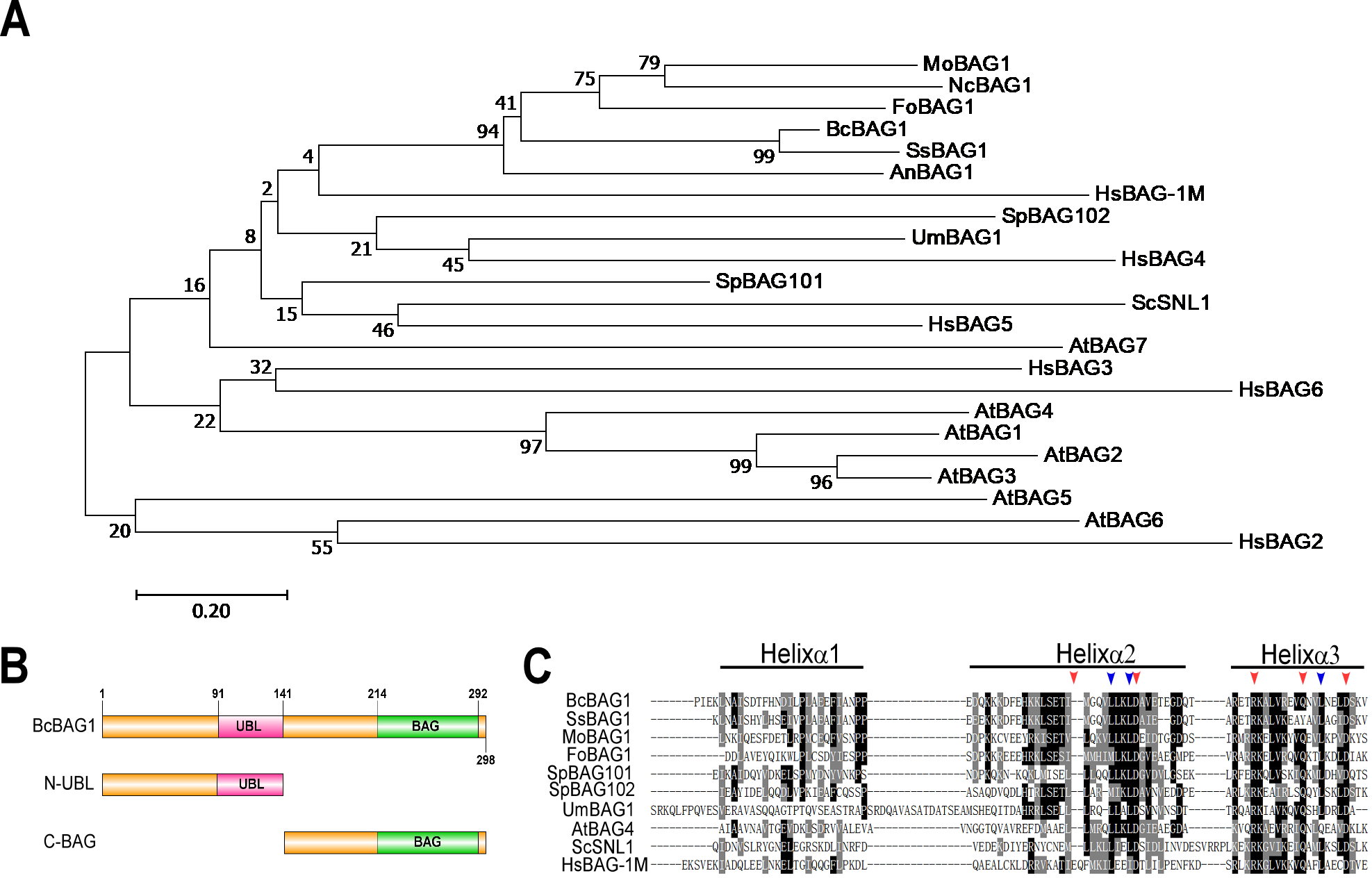

Supplement: Figure S1 — Phylogenetic and sequence analysis of BAG1 in Botrytis cinerea (BcBAG1). (A) The phylogenetic tree of BAG proteins. Evolutionary analyses were conducted in MEGA7.0. The evolutionary history was inferred by using a neighbor-joining method based on the amino acid sequences. The numbers at nodes inferred the percentage of their occurrence in 10,000 bootstrap replicates. Species names and GenBank accession numbers of each sequence are represented as follows: SsBAG1 (Sclerotinia sclerotiorum, XP_001591798.1); MoBAG1 (Magnaporthe oryzae, XP_003710309.1); FoBAG1 (Fusarium oxysporum f. sp. Lycopersici, XP_018239181.1); NcBAG1 (Neurospora crassa, XP_961586.1); AnBAG1 (Aspergillus nidulans, XP_661815.1); UmBAG1 (Ustilago maydis, KIS67500.1); SpBAG101 (Schizosaccharomyces pombe, NP_596760.1); SpBAG102 (Schizosaccharomyces pombe, NP_595316.1); ScSNL1 (Saccharomyces cerevisiae, KZV10602.1); HsBAG-1M (Homo sapiens, NP_001336215.1); HsBAG2 (Homo sapiens, NP_004273.1); HsBAG3 (Homo sapiens, NP_004272.2); HsBAG4 (Homo sapiens, NP_004865.1); HsBAG5 (Homo sapiens, NP_001015049.1); HsBAG6 (Homo sapiens, P46379.2); AtBAG1 (Arabidopsis thaliana, NP_200019.2); AtBAG2 (Arabidopsis thaliana, NP_568950.2); AtBAG3 (Arabidopsis thaliana, NP_196339.1); AtBAG4 (Arabidopsis thaliana, NP_190746.2); AtBAG5 (Arabidopsis thaliana, NP_172670.2); AtBAG6 (Arabidopsis thaliana, AEC10664.1); AtBAG7 (Arabidopsis thaliana, NP_201045.1). (B) Schematic diagram of BcBAG1. Purple red and green boxes indicate the UBL and BAG domain of BcBAG1, respectively. (C) Sequence alignment of the conserved BAG domain from different organisms. Three predicted helixes labeled on the top. Conserved residues which involving in binding of BAG protein to Hsc70 ATPase domain in human HsBAG-1M are indicated by red arrow, and residues critical to packing interactions are highlighted by blue arrow. [file Image_1.TIF]

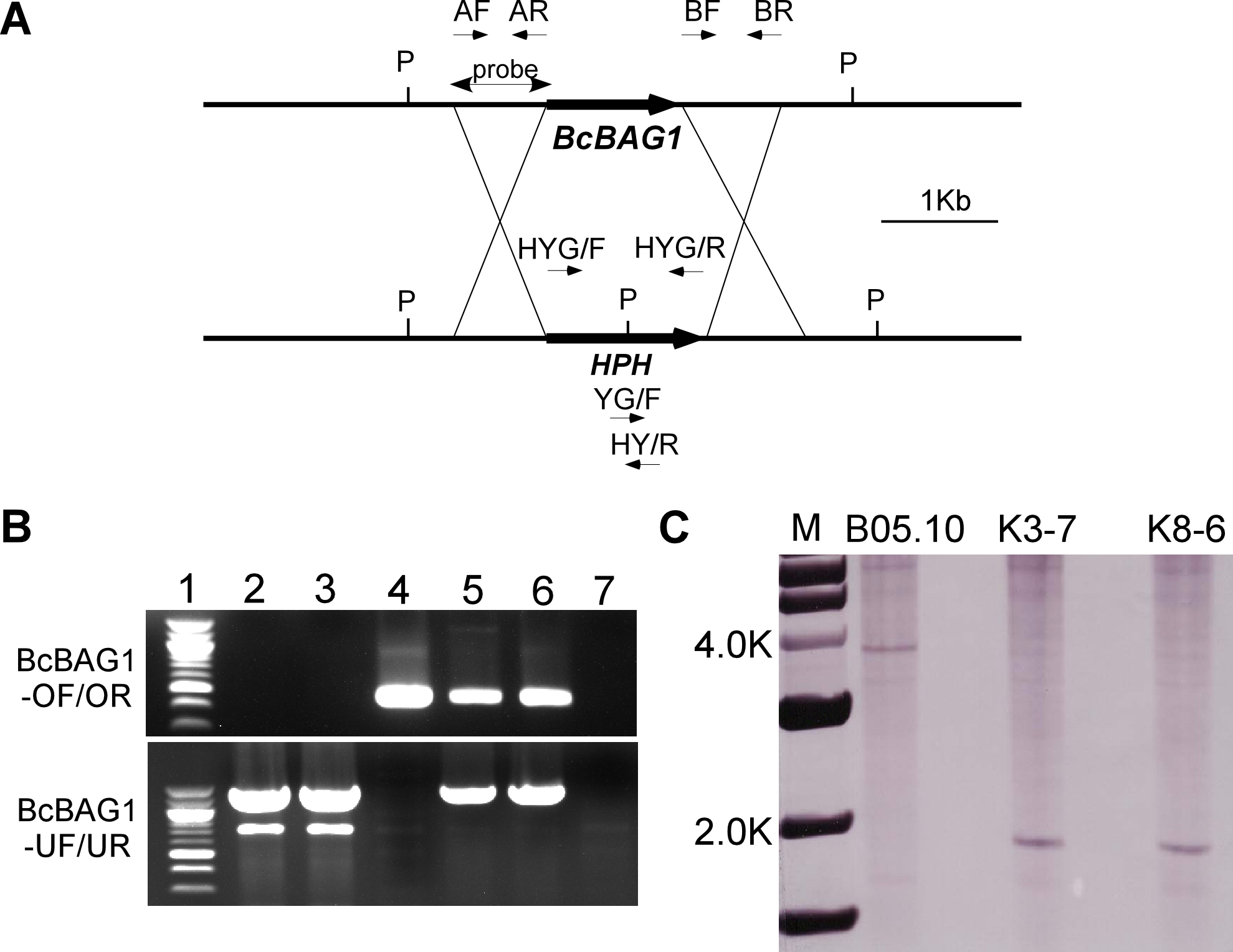

Supplement: Figure S2 — Sketch of gene deletion and identification of deletion mutants and complemented transformants. (A) Diagram of targeted gene replacement. (B) RT-PCR confirmation. Lane 1:100 bp DNA Ladder (NEB); lanes 2 and 3: K3-7 and K8-6, respectively; lane 4: B05.10; lanes 5 and 6: complemented transformants; lane 7: PCR negative control. (C) Southern blotting confirmation. Total genomic DNAs were digested by PvuI, and a DNA fragment in the upstream of 5′ terminus of BcBAG1 was selected and labeled as the probe shown in panel (A). M: 1 kb DNA Ladder (NEB). [file Image_2.TIF]

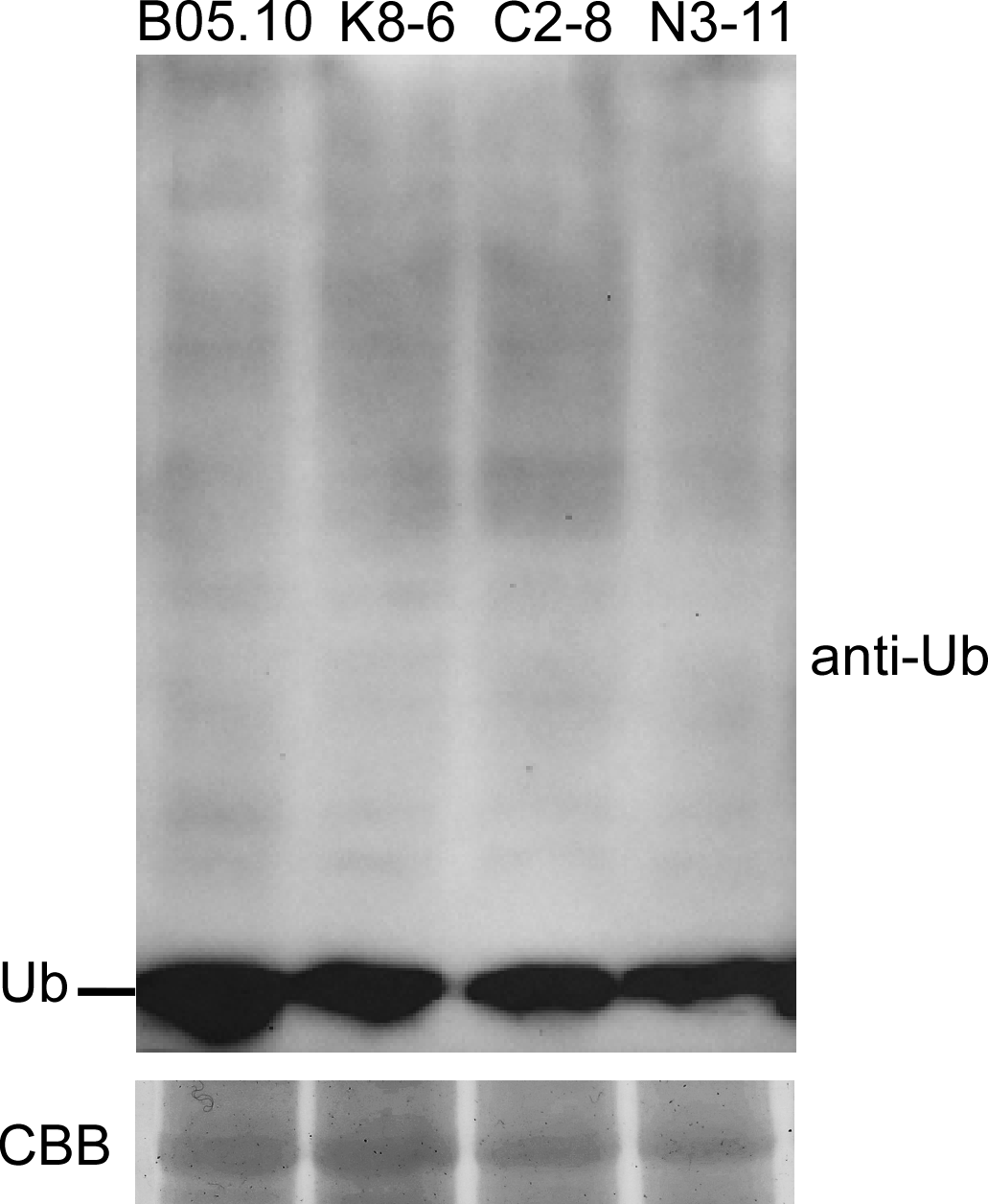

Supplement: Figure S3 — Disruption of BcBAG1 does not affect protein ubiquitination. Total protein extracts from corresponding strains was western blotted with (upper panel) an anti-Ub antibody (P4D1) and stained with Coomassie brilliant blue (lower panel) as the loading control. B05.10: the wild-type strain; K8-6: ΔBcbag1 mutant lines; C2-8 (C-terminus of BcBAG1) and N3-11 (N-terminus of BcBAG1). [file Image_3.TIF]

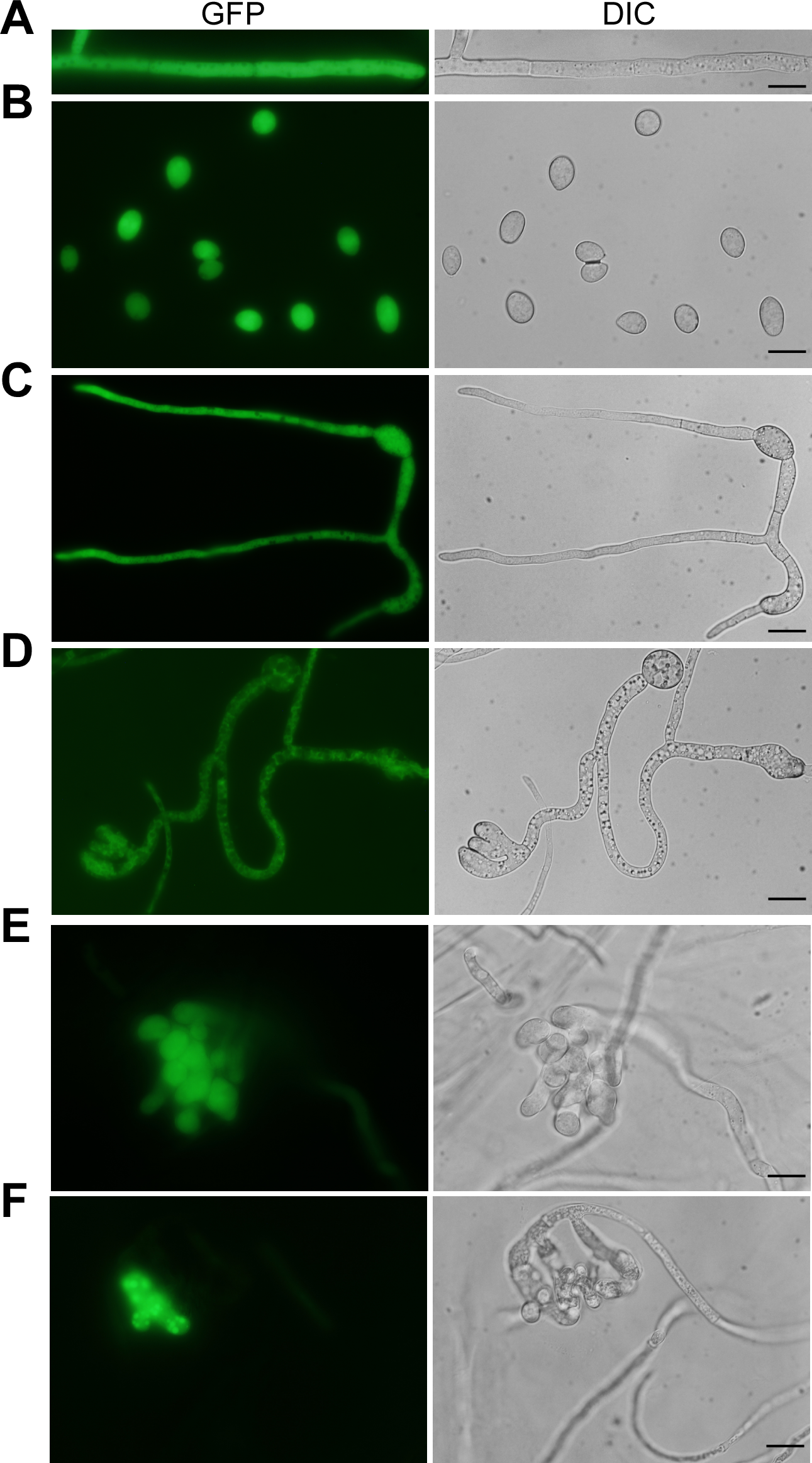

Supplement: Figure S4 — Subcellular localization of BcBAG1. GFP-BcBAG1 was overexpressed in the ΔBcbag1 mutant (OG2-7) and the fluorescence for BcBAG1 localization at different stages was visualized by confocal microscopy. (A) Vegetative hyphae; (B) conidia; (C,D) conidia on hydrophobic glass slides for 12 and 24 h, respectively. (E,F) Both are conidia on onion epidermal cells for 24 h and 48 h, respectively. Scale bars: 20 μm. [file Image_4.TIF]
