# Supplementary material for: Cytoprotective Co-chaperone BcBAG1 Is a Component for Fungal Development, Virulence, and Unfolded Protein Response (UPR) of Botrytis cinerea
Source: Front Microbiol. 2019 Apr 9;10:685. doi: 10.3389/fmicb.2019.00685 (PMC6467101; doi:10.3389/fmicb.2019.00685)
Supplement: Table S1 — Wild-type and mutant strains of Botrytis cinerea used in this study. [file Table_1.DOCX]

**Table 1.** Wild-type and mutant strains of *Botrytis cinerea* used in this study.

| **Strain** | **Genotype description** | **References** |
| --- | --- | --- |
| B05.10 | Isolate from *Vitis vinifera*, MAT1-1 | ([Quidde *et al.*, 1999](#_ENREF_53)) |
| K3-7 | B05.10, *Δbcbag1*::hph, homokaryon | This work |
| K8-6 |  | This work |
| *BcBAG1-Com* | B05.10, *bcbag1*::hph, BcBAG1::nat1, heterokaryon | This work |
| C2-8 | B05.10, *bcbag1*::hph, BcBAG1^142-298^::nat1, heterokaryon | This work |
| N3-11 | B05.10, *bcbag1*::hph, BcBAG1^1-141^::nat1, heterokaryon | This work |
| OG2-7 | B05.10, P*oliC*::BcBAG1-gfp::hph, heterokaryon | This work |
