# Supplementary material for: Cytoprotective Co-chaperone BcBAG1 Is a Component for Fungal Development, Virulence, and Unfolded Protein Response (UPR) of Botrytis cinerea
Source: Front Microbiol. 2019 Apr 9;10:685. doi: 10.3389/fmicb.2019.00685 (PMC6467101; doi:10.3389/fmicb.2019.00685)
Supplement: Table S2 — Oligonucleotide primers used in this study. [file Table_2.DOCX]

**Table S2.** Oligonucleotide primers used in this study.

| **Primer** | **Sequence (5’-3’)** | **Relevant characteristics** |
| --- | --- | --- |
| BcBAG1-AF | AACGGGAGAGTTTGGAGT | PCR primers to amplify BcBAG1 upstream fragment for the construction of BcBAG1 deletion mutant |
| BcBAG1-AR | TTGACCTCCACTAGCTCCAGCCAAGCCATGGCAGTAGACAGGGTG |  |
| BcBAG1-BF | GAATAGAGTAGATGCCGACCGCGGGTTCGGATTCCTTCTGTATTGAG | PCR primers to amplify BcBAG1 downstream fragment for the construction of BcBAG1 deletion mutant |
| BcBAG1-BR | CTGATGGCAGATAATAAGGC |  |
| HYG/F | GGCTTGGCTGGAGCTAGTGGAGGTCAA | PCR primers for amplification of hygromycin resistance gene (HPH) |
| HYG/R | AACCCGCGGTCGGCATCTACTCTATTC |  |
| YG/F | GATGTAGGAGGGCGTGGATATGTCCT |  |
| HY/R | GTATTGACCGATTCCTTGCGGTCCGAA |  |
| BcBAG1-OF | AGTCGTTGGGGAAATAGTTG | PCR primers for identification of BcBAG1 deletion transformants |
| BcBAG1-OR | CGTTGTGGAAGGTATCTGAG |  |
| BcBAG1-UF | CAACACAGAGCGGTGATAGC |  |
| BcBAG1-UR | GTCCATCACAGTTTGCCAGT |  |
| BcBAG1-CF | GGACTAGTTCTGCTCCGAACAACATGC | PCR primers to amplify full cDNA sequence of BcBAG1 gene for complementation |
| BcBAG1-CR | CATGCCATGGTAGAAGCCCAATCATACCCAG |  |
| BcBAG1-GF | CATGCCATGGATGAGTCGTTGGGGAAATAGTT | PCR primers to amplify BcBAG1 full fragment for GFP construction of pNAH-OGG |
| BcBAG1-GR | CATGCCATGGCTTCTGCACCTGGAACCTTTG |  |
| THR1-ex-F | TCTGGTTCTAAGGGTGCCATT | PCR primers to amplify THR1 downstream fragment for the expression levels analysis |
| THR1-ex-R | CCTTTCCGTTAACCCATTCA |  |
| BcActin-F | CGAGCAAGAAATCCAAAC | PCR primers to amplify Actin fragment for RT-PCR |
| BcActin-R | GAACCACCAATCCAGACG |  |
| β-tubulin-F | ACCGTTCCAGAGTTGACTCAA | PCR primers to amplify β-tubulin downstream fragment for the expression levels analysis |
| β-tubulin-R | GCAAGAAAGCCTTTCTTCTGA |  |
| UBL-F | CATGCCATGGATGAGTCGTTGGGGAAATAGT | PCR primers to amplify N teminal (UBL domain) of BcBAG1 gene for complementation |
| UBL-R | CATGCCATGGCACAATGCAAAGAATTTCCG |  |
| BD-F | CATGCCATGGATGGGAGATGCAGCCACG | PCR primers to amplify C teminal (BAG domain) of BcBAG1 gene for complementation |
| BD-R | CATGCCATGGCTTCTGCACCTGGAACCTTTG |  |
| BcBIP1ADF | CGGAATTCATGGCCAGTGGAAACAGATC | PCR primers for AD-BcBIP1 constructs, BcBIP1 (BC1G_04390) |
| BcBIP1ADR | CGGGATCCCTACAACTCATCGTGGCCCT |  |
| BcSSC1ADF | TCCCCCGGGTATGTTTGCTTCCAGAATTTCC | PCR primers for AD-BcSSC1 constructs, BcSSC1 (BC1G_11661) |
| BcSSC1ADR | CGGGATCCTCAAGGCTTCTTCTCTCCGT |  |
| BcSKS2ADF | CGGAATTCATGGCCGACGAAGTTTAC | PCR primers for AD-BcSSC1 constructs, BcSKS1 (BC1G_10846) |
| BcSKS2ADR | CGAGCTCTTAACATACTGGAACAGTTTGAC |  |
| BcSSA1ADF | TCCCCCGGGTATGGCTCCAGCTATTGGT | PCR primers for AD-BcSSC1 constructs, BcSSA1 (BC1G_06164) |
| BcSSA1ADR | CGGGATCC TTAGTCGACCTCCTCGATCT |  |
| BcPSS1ADF | TCCCGGCCATGGAGGCCATGTCTGTCGTCGGTGTAGATT | PCR primers for AD-BcSSC1 constructs, BcPSS1 BC1G_09769 |
| BcPSS1ADR | TCCCCCGGGTTACTTCTCCTCAACCTCTGGC |  |
| BcLHS1ADF | TCCCCCGGGTATGGCTCTTTTGAAGAGTCCT | PCR primers for AD-BcSSC1 constructs, BcLHS1 (BC1G_12543) |
| BcLHS1ADR | CGAGCTCCTACAATTCTTCATGCTTAACCTT |  |
| BcBAG1-BDF | TCCCCCGGGTATGAGTCGTTGGGGAAATAGT | PCR primers for BD-BcBAG1 constructs |
| BcBAG1-BDR | CGGGATCCTTATTCTGCACCTGGAACCTT |  |
| BcHAC1-RTF | CCCCTTCAATGGAATCAA | PCR primers to amplify BcHAC1 fragment for RT-PCR |
| BcHAC1-RTR | CGTCGCATCCAAAAGAGG |  |
| BcBAG1-QF | ACGAATTCGCAAATCGAAGA | PCR primers to amplify BcBAG1 fragment for the expression levels analysis |
| BcBAG1-QR | CGTTGTGGAAGGTATCTGAG |  |
| BcIRE1-QF | ATTAGTCGGGACGGGTAG | PCR primers to amplify BcIRE1 fragment for the expression levels analysis |
| BcIRE1-QR | TCAACGAGGCGGGACAT |  |
| BcBIP1-QF | AAAGATGAAGGAAGTCGCCG | PCR primers to amplify BcBIP1 fragment for the expression levels analysis |
| BcBIP1-QR | ACGGAGGACATTCAAGCC |  |
| BcBAG1-1F | CATGCCATGGTCTGCTCCGAACAACATGC | PCR primers for amplification of full cDNA sequence of BcBAG1 gene for sequencing and construction of pCB302ES- BcBAG1 |
| BcBAG1-1R | GAAGGCCTTTCTGCACCTGGAACCTTTG |  |
| EF1a-F | TGCTGTCCTTATCATTGACTCCACCAC | PCR primers to amplify EF1a fragment for the expression levels analysis |
| EF1a-R | TTGGAGTACTTGGGGGTAGTGGCATC |  |
