# Supplementary material for: Cytoprotective Co-chaperone BcBAG1 Is a Component for Fungal Development, Virulence, and Unfolded Protein Response (UPR) of Botrytis cinerea
Source: Front Microbiol. 2019 Apr 9;10:685. doi: 10.3389/fmicb.2019.00685 (PMC6467101; doi:10.3389/fmicb.2019.00685)
Supplement: Table S3 — Similarity of full length and BAG domain amino acid sequence between BcBAG1 and other BAGs. [file Table_3.DOCX]

**Table S3.** Similarity of full length and BAG domain amino acid sequence between BcBAG1 and other BAGs.

| **Similarity**  **Protein** | **Full length** | **BAG domain** |
| --- | --- | --- |
| SsBAG1 | 86.53% | 86.07% |
| MoBAG1 | 56.37% | 62.02% |
| FoBAG1 | 51.67% | 58.97% |
| NcBAG1 | 47.65% | 53.75% |
| AnBAG1 | 45.30% | 62.82% |
| SpBAG101 | 37.94% | 45.56% |
| SpBAG102 | 29.61% | 47.43% |
| ScSNL1 | 30.81% | 46.91% |
| UmBAG1 | 27.34% | 33.33% |
| AtBAG1 | 21.47% | 44.74% |
| AtBAG2 | 22.80% | 40.78% |
| AtBAG3 | 21.14% | 43.42% |
| AtBAG4 | 23.79% | 50.66% |
| AtBAG5 | 16.74% | 44.44% |
| AtBAG6 | 15.43% | 44.59% |
| AtBAG7 | 23.48% | 40.74% |
| HsBAG-1M | 23.72% | 35.80% |
| HsBAG2 | 18.48% | 23.45% |
| HsBAG3 | 16.44% | 16.90% |
| HsBAG4 | 19.46% | 41.42% |
| HsBAG5 | 23.15% | 44%,33.33%,40.62% |
| HsBAG6 | 15.43% | 30.86% |
